# Control of *Blumeria graminis* f. sp. *hordei* on Barley Leaves by Treatment with Fungi-Consuming Protist Isolates

**DOI:** 10.1007/s00284-023-03497-5

**Published:** 2023-10-23

**Authors:** Julia Sacharow, Elnaz Salehi-Mobarakeh, Stefan Ratering, Jafargholi Imani, Alessandra Österreicher Cunha-Dupont, Sylvia Schnell

**Affiliations:** 1 Institute of Applied Microbiology, IFZ, Justus-Liebig-University, Giessen, Germany; 2 Institute of Phytopathology, IFZ, Justus-Liebig-University, Giessen, Germany

## Abstract

The obligate biotrophic fungal pathogen *Blumeria graminis* causes the powdery mildew disease of cereals, which results in large crop losses*.* Control of *B. graminis* in barley is mainly achieved by fungicide treatment and by breeding resistant varieties. Vampyrellid amoebae, just like mycophagous protists, are able to consume a variety of fungi. To reveal the impact of some selected fungus-consuming protists on *Blumeria graminis* f. sp. *hordei* (*Bgh*), and to evaluate the possibility of using these protists as biological agents in the future, their feeding behaviour on *B. graminis* spores on barley leaves was investigated. An experiment was carried out with five different protist isolates (*Leptophrys vorax*, *Platyreta germanica*, *Theratromyxa weberi* U 11, *Theratromyxa weberi* G7.2 and *Acanthamoeba castellanii*) and four matched controls, including the food sources of the cultures and the medium. Ten-day-old leaves of barley (*Hordeum vulgare* cv. Golden Promise) were first inoculated with *Blumeria graminis* (f. sp. *hordei* race A6) spores, then treated with protists and fungal colonies on the leaf surfaces were counted under the microscope after 5 days. The isolates *L. vorax*, *P. germanica,* and *T. weberi* U11 did not show a significant reduction in the number of powdery mildew colonies whereas the isolates *T. weberi* G7.2 and *A. castellanii* significantly reduced the number of powdery mildew colonies on the leaf surfaces compared to their respective controls. This indicates that these two isolates are capable of reducing *B. graminis* colonies on barley leaves and are suitable candidates for further investigation for possible use as biological agents. Nevertheless, the susceptibility to dryness and the cell division rate should be considered during the optimisation of the next steps like application procedure and whole plant treatment.

**Supplementary Information:**

The online version contains supplementary material available at 10.1007/s00284-023-03497-5.

## Introduction

The crop barley was domesticated around 7800–7500 B.C. and is nowadays the fourth most important economically cultivated crop, besides wheat, rice and maize [1, 2]. The airborne obligate biotrophic fungus *Blumeria graminis* f. sp. *hordei* causes powdery mildew on barley, which is a major and damaging disease for cereal production in northern Europe [3]. It has a mixed reproductive system, with a sexual cycle during the growing season and an asexual cycle during the epidemic phase and is classified as a high-risk pathogen due to its large population size and high potential for adaptation [4, 5]. Growing resistant barley varieties is a good economically and environmentally friendly way to control the disease [6]. These resistant varieties are highly effective against the fungus, but research is time consuming, and some varieties have shown lower yield performance [6, 7]. Furthermore, the plant’s high anti-fungal efficacy only lasts for a short period of time, because of the production of various *B. graminis* f. sp. *hordei* virulence genes. During fungal sexual reproduction, new combinations of alleles are produced and distributed through asexual reproduction. These new combinations render the resistant plant varieties ineffective within a few years [6, 8]. Due to short-lived resistances, *B. graminis* f. sp. *hordei* is controlled by a combination of resistant varieties and fungicides, such as demethylation inhibitor (DMI) fungicides. In addition to the negative environmental impact of fungicides, such as run-off from agricultural land and accumulation in surrounding water bodies, this practice leads to the development of fungicide-resistant races of *B. graminis* f. sp.* hordei*. The appearance of resistant haplotypes can increase from 0 to 90% within two years and cause major economic problems for the barley production [9]. This calls for new, sustainable and environmentally friendly approaches, such as the use of biological control agents. Biological control agents are already being researched and used in several areas [10–12].

Vampyrellid amoebae from marine, freshwater and soil environments can feed on a wide range of different eukaryotes such as spores of fungi, diatoms and nematodes. They have the ability to engulf their prey whole or to perforate the cell wall [13]. Their feeding strategy depends on the prey species: *Sericomyxa perlucida*, for example, is able to engulf small diatoms and to feed on larger species by extraction of the protoplast with an invading pseudopodium [14]. The same was observed for *Leptophrys vorax*. *L. vorax* is able to engulf single algae cells, to engulf algae colonies or to invade them [15]. The order Vampyrellida includes several genera [16], and some examples are *Leptophrys* [17], *Platyreta* [18] and *Theratromyxa* [19]. Knowledge on the interactions between vampyrellid amoebae and fungi and nematodes raised interest on their use in agricultural pest control as biological control agents of soil-borne plant pathogens, such as the larvae of the nematode *Heterodera rostochiensis* [20]. It should be noted that the identification of the organisms may not be correct due to the imprecise methods of determination at the time. Soil inoculated with cysts of the nematode *H. rostochiensis* and the vampyrellid amoeba *Theratromyxa* showed that the amoebae was able to feed on the cysts, but the slow spread rate, the susceptibility to drought and the non-specific consumption of prey by the protists indicated that they were not suitable as biological agents [21]. The feeding behaviour of *Theratromyxa weberi* on the prey nematode *Aphelenchoides rutgersi*, which is a common pathogen of citrus plants, showed that the vampyrellid amoebae was able to engulf the prey in less than 3 h [22]. *Arachnula impatiens* and *L. vorax* were also tested on three different plant-parasitic nematodes, *Meloidogyne incognita*, *Aphelenchoides besseyi* and *Helicotylenchus dihystera*. The vampyrellid amoebae were able to feed on the three nematodes, and *Arachnula impatiens* was able to empty the larva of *Meloidogyne incognita* within 2–3 h, while *L. vorax* encysted the larva for 12–24 h [23]. Vampyrellid amoebae are also capable of lysing conidia and fungal spores [24]. *Cochliobolus sativus* inoculated with vampyrellid amoebae in different soils showed perforations and lysis of conidia [25]. Under cultural conditions, the amoebae *Arachnula impatiens* was able to perforate and lyse hyphae and chlamydospores of the fungus *Phytophthora cinnamomi* [25]. It was also reported that vampyrellid amoebae were able to perforate the hyphae of the plant pathogen *Gaeumannomyces graminis,* which causes the plant disease Take-all [26]. Opposing results were observed on the feeding behaviour of an unidentified vampyrellid amoebae on the fungi *G. graminis* var. *tritici*, *Cochliobolus sativus* and *P. cinnamomi* [27]. The unidentified vampyrellid amoebae showed no mycophagous behaviour and no perforations in the scanning electron microscope images. Nevertheless, it is suggested that vampyrellid amoebae have considerable potential for pathogen control through regulation of the rhizosphere microflora, leading to possible suppression of disease and favourable conditions for plant growth [28]. In addition to vampyrellid amoebae, genera like *Acanthamoeba*, which was previously thought to be a bacterivorous protist, were shown to be able to feed on various yeasts, but not on the hyphae of the fungus *Fusarium culmorum* [29], indicative of the existence of facultative mycophagous protists.

The aim of this study was to test for possible fungus-eating protists candidates to be used in further studies as biological control agents in agricultural crop production against the spores of fungal pathogens. Infected barley leaves were treated with different vampyrellid amoebae or mycophagous amoebae to observe their feeding on the spores of the plant pathogen *Blumeria graminis* f. sp. *hordei* race A6.

## Material and Methods

### Protist and Fungus Cultures

Five different amoeboid protists were selected for the experiment: *Leptophrys vorax*, *Platyreta germanica*, *Theratromyxa weberi* U11, *Theratromyxa weberi* G7.2 and *Acanthamoeba castellanii. Acanthamoeba castellanii* (strain CCAP 1534/3) was ordered from CCAP—Culture Collection of Algae and Protozoa (Oban, United Kingdom). *Leptophrys vorax* (strain CCAC 3424—Culture Collection of Algae at the University of Cologne (Cologne, Germany)), *Platyreta germanica* (strain CCAP 2559/1), *Theratromyxa weberi* U11 and *Theratromyxa weberi* G7.2 (strain CCAP 2573/1) were provided by Sebastian Hess (Institute for Zoology, University of Cologne, Cologne, Germany). *L. vorax*, *P. germanica*, *T. weberi* U11 and *T. weberi* G7.2 were fed with the yeast *Saccharomyces cerevisiae* H15, also provided by Sebastian Hess. The yeast was grown on malt extract agar (Carl Roth, Germany) at 25 °C in a climatic chamber. *A. castellanii* was fed with an unidentified mixture of *Escherichia coli* and various bacteria, obtained from the CCAP.

*Blumeria graminis* f. sp. *hordei* race A6 (hereafter: *Bgh*A6) was grown and maintained on small plants of the sensitive Scottish malting spring barley cv Golden Promise in a climate chamber at 22 °C/18 °C in a day/night cycle, 60% relative humidity and a 16 h photoperiod with 240 µmol m^−2^ s^−1^ photon flux density.

### *Blumeria graminis* Spore Consumption in Liquid Cultures

In a first experiment, the ability of the protists to interact with and to consume *Bgh*A6 spores was tested at room temperature in half-strength Waris-H medium [30]. For the medium 0.50 ml of the stock solutions (stock solution in 1000 ml (final concentration in the media): HEPES 238 g (1 mM); KNO_3_ 100 g (1 mM); MgSO_4_ × 7 H_2_O 20 g (81.1 µM); (NH_4_)_2_HPO_4_ 20 g (0.15 mM); Ca(NO_3_)_2_ × 4 H_2_O 100 g (0.42 mM)) and 50 ml soil extract were added to 947.50 ml dH_2_O. For the soil extract, 10 g of grassland soil was mixed with 120 ml dH_2_O, boiled and stirred for 10 min on a hot plate. After the mixture was cooled down, it was filled in tubes (Sarstedt AG & Co. KG, Germany) and centrifuged at 1000*g* (Megafuge 1.OR, Thermo Fischer Scientific, USA) for 10 min. The supernatant was filtered through a 25 µm, 3 µm and 0.2 µm series of membrane filters (Whatman, United Kingdom), and the remaining filtrate was adjusted to 100 ml with dH_2_O (modified after the protocol of Central Collection of Algal Cultures). The medium was adjusted (Mettler-Toledo, Germany) to pH 7 with NaOH and autoclaved (HV-25L, HMC-Europe GmbH, Germany) at 121 °C for 20 min.

After counting the protist culture with a counting chamber (Neubauer improved, Hecht-Assistant, Germany) five millilitre of the 10^5^ ml^−1^ protist culture was added to a large Petri dish (Ø 14.5 cm, 83.3903, Sarstedt AG & Co. KG, Germany) containing 100 ml of the medium, and then a barley plant infected with *Bgh*A6 was shaken over the open plate and the spores on the surface of the liquid culture were mixed by pipetting. After 1 week, the cultures were examined with the microscope under a 630× magnification (DM 1000 LED, Leica, Germany) to check for the consumption of the fungus spores by the protists. Microscopy was repeated after two, three and four weeks. Microscopy images were further processed and assembled using Pixlr Inmagine Lab (Pixlr X, Sweden), Corel PaintShop (Pro 2023, Canada) and ImageJ version 1.47 [31].

### Infection of the Leaves with *Blumeria graminis* and Treatment with the Amoeboid Protists

**Fig. 1:**
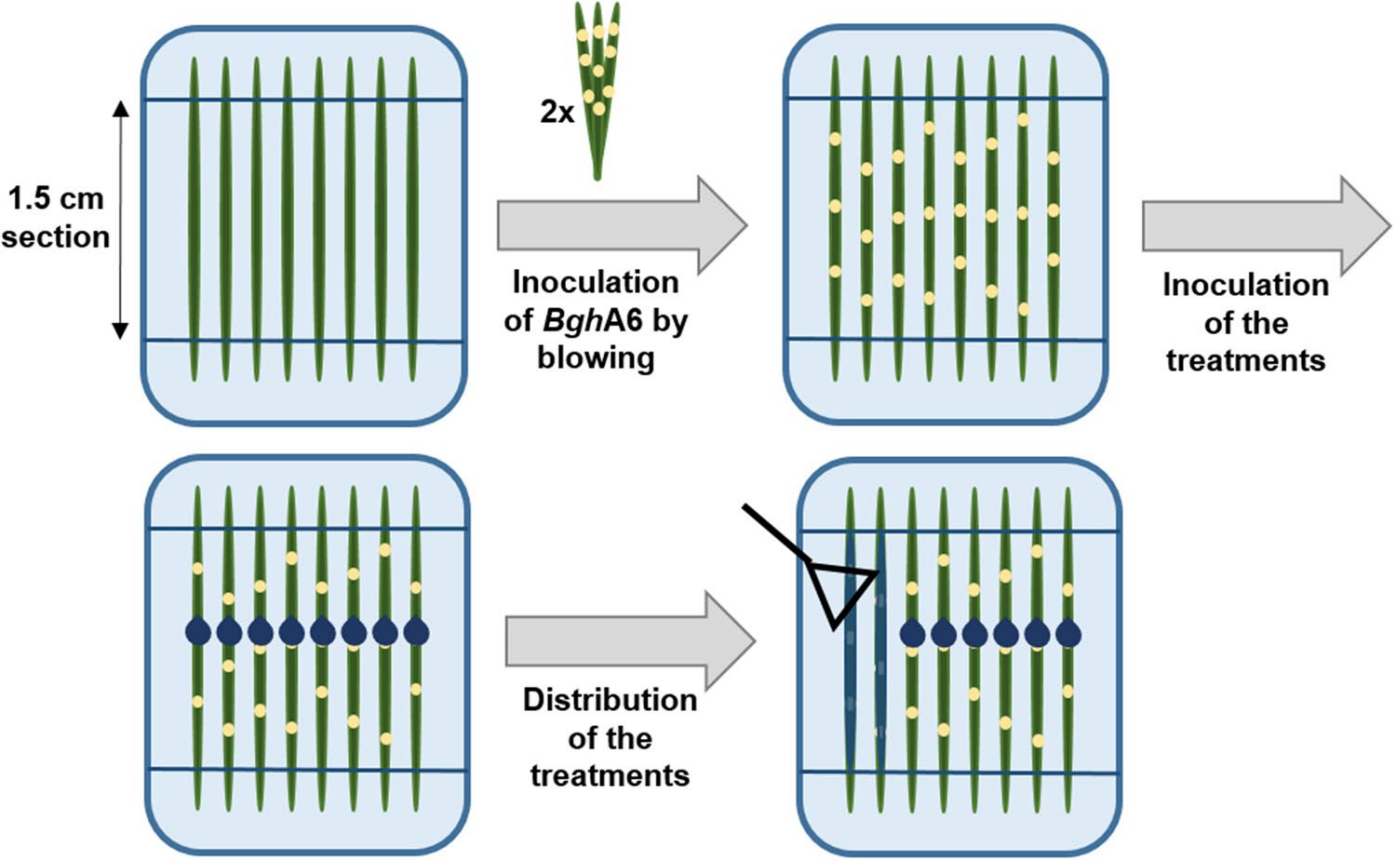
Overview of the inoculation steps in the experiment. Starting with the first true leaves of the Scottish malting spring barley cv Golden Promise on water agar with benzimidazole. Following by the inoculation with *Blumeria graminis* f. sp. *hordei* race A6 spores by blowing and the distribution of the treatments using a pipette and Drigalski spatula

The sensitive Scottish malting spring barley cv Golden Promise was used for the experiment. Ten Golden Promise seeds were sown in a small pot (Ø 6 cm) with a fertilized soil (type T, Fruhstorfer Erde, Hawita Gruppe, Germany) and grown in a climate chamber for ten days (conditions described above). Leaf segments were collected with sterile scissors and laid on 0.6% (wt/vol) water agar containing benzimidazole (0,0044%, Merck Schuchardt oHG, Germany). In detail, 6 g of agar (Roth, Germany) was added to 960 ml dH_2_O, autoclaved as described above and cooled. Benzimidazole solution (40 ml) was added to the water agar, shaken gently and poured into square Petri dishes. The leaf segments were fixed with sterile sticks in the square Petri dishes (Sarstedt AG & Co. KG, Germany) and inoculated with fresh spores of *Bgh*A6 (Fig. 1) in an inoculation tower. Specifically, six plates were opened and placed on the ground. A closed settling tower was placed above the plates and inoculated two times with three infected leaves by blowing spores from infected leaves inside the tower. The spore concentration was approximately 10^4^ ml^−1^, determined by picking six similarly infected leaves, putting them in a glass bottle (Schott, Germany) with 100 ml half-strength Waris-H medium, washing the spores of by putting the bottle in an overhead shaker (Heidolph instruments GmbH & Co.KG, Germany) for 10 min with 1000 RPM and counting the spore concentration with the counting chamber (Neubauer improved). After the blowing, to ensure an even distribution of the spores, a metal distributor was swung into the tower, and the tower was closed again for 10 min, to allow the spores to settle down [3, 32]. Then the Petri dishes were closed and left at the climate chamber for 60 min for the initial contact. Besides the tower inoculation, some other methods (inoculation in suspension, inoculation with a pipette and a Drigalski spreader and inoculation by suspension spraying) were tested in a pre-experiment. The results (Tab. S1) showed that these other methods were not suitable for this experiment.

One hour after the first inoculation, a second inoculation was made with the fungal consuming cultures. For the experiment, the protists were cultured (several replicates) one month before (as described above). *L. vorax*, *P. germanica*, *T. weberi* U11 and *T. weberi* G7.2 were fed with *S. cerevisiae* and *A. castellanii* was fed with a mixed bacterial culture. During the cultivation, the cultures were checked regularly by microscopy, fed with fresh prey and the plates were filled up with fresh half-strength Waris-H, if necessary. One day before the experiment, the cultures were examined under the microscope and cultures that showed good growth and high quality (high amount of trophozoites and several cysts) were selected and concentrated by centrifugation at 1000*g* for 10 min. The supernatant from *A. castellanii* culture was retained for a control treatment. All concentrated cultures were individually stored at room temperature in autoclaved glass bottles (one bottle per treatment). The experiment consisted of nine different treatments (Table 1). The first five treatments, i.e. the fungus-consuming cultures in half-strength Waris-H medium spread over *Bgh*A6-infected leaves and four control treatments. The control solutions consisted of the food sources (6) and (7) in half-strength Waris-H medium spread over *Bgh*A6-infected leaves, the medium (8) spread over *Bgh*A6-infected leaves and unaffected *Bgh*A6 spores (9) without a second inoculation. On each leaf of the plate, 1 ml of the protist culture 10^5^ ml^−1^_,_ determined with the counting chamber, was dripped with a pipette and spread over the surface of the central 1.5 cm section using a sterile Drigalski spreader. Five Petri dishes were prepared per treatment, with eight leaves in each Petri dish. The four control treatments (Table 1) were treated in the same way. For the first control (6), the number of remaining *S. cerevisiae* cells in the protist cultures was counted using the counting chamber. Based on the count results, a culture of *S. cerevisiae* in half-strength Waris-H with a concentration of 10^6^ ml^−1^ was prepared, and the leaves were treated as described before. For the control (7), the remaining bacterial cells in the *A. castellanii* culture were counted with the counting chamber and the supernatant adjusted to the results of 10^7 ^bacteria ml^−1^. The inoculated plates were kept in the climate chamber for 5 days (same conditions as described previously) and covered with paper to create an environment without artificial light. This procedure was tested in a pre-experiment, where half-strength Waris-H medium was applied before and after the inoculation of *Bgh*A6 spores. It showed that the application of the medium before the *Bgh*A6 spores resulted in a slower colony development, with smaller colonies, in comparison to the chosen application method (Table S2).

**Table 1:** Overview of the treatments and their individually suitable controls

Treatment	Suitable controls
(1) *L. vorax*	(6) Liquid culture of *S. cerevisiae*, (8) Half-strength Waris-H medium and (9) Only *Bgh*A6
(2) *P. germanica*	(6) Liquid culture of *S. cerevisiae*, (8) Half-strength Waris-H medium and (9) Only *Bgh*A6
(3) *T. weberi* U11	(6) Liquid culture of *S. cerevisiae*, (8) Half-strength Waris-H medium and (9) Only *Bgh*A6
(4) *T. weberi* G7.2	(6) Liquid culture of *S. cerevisiae*, (8) Half-strength Waris-H medium and (9) Only *Bgh*A6
(5) *A. castellanii*	(7) Bacterial supernatant of *A. castellanii*, (8) Half-strength Waris-H medium and (9) Only *Bgh*A6

### Evaluation of the Powdery Mildew Colonies and Preparation of Microscopy Photographs

Four plates of each treatment were randomly selected and used for the evaluation. The central parts of the leaves were counted individually by placing a metal stencil with a 1 cm^2^ opening on the leaf. The powdery mildew colonies in this area were counted under a stereomicroscope (SZ-PT, Olympus, Japan).

Some of the infected leaves were used for subcultures, to see if the protists have survived the inoculation process and incubation time. Small pieces of the agar and from the central parts of the inoculated leaves were cut out with a scalpel (B. Braun, Germany). The agar and the leaf pieces were placed separately in Petri dishes and filled with half-strength Waris-H and *S. cerevisiae.* They were observed under the microscope after two days at room temperature.

### Spraying of the Amoeboid Protists

Two parallel cultures of *A. castellanii* were used for a comparison of different spraying methods to investigate the potential for spraying protists on infected leaves. Only *A. castellanii* was chosen for this small side experiment, because it is smaller, more stable and has a fast reproduction rate in comparison to the other protists used in this experiment. The *A. castellanii* cultures were examined under a microscope, then one whole culture was sprayed with a brown glass spray bottle (Carl Roth, Germany) and the other culture was sprayed with a softer test tube sprayer (Duran, Germany) into a fresh Petri dish. After spraying, the cultures were left at room temperature for two hours and the cell shape was examined again under the microscope.

### Statistical Analysis

Graphics and statistical analysis were performed using R version 4.0.3 [33]. Data were tested for normal distribution using the Shapiro–Wilk’s test [34], analysed using a non-parametric Kruskal–Wallis test [35] and a post hoc pairwise Wilcoxon rank sum test [36] (*P* < 0.05). To calculate the pairwise comparisons between the group levels, the *P*-values were multiplied by the number of comparisons by using the Benjamini & Hochberg adjustment method [37]. The package ggplot2 was used for the graph [38].

## Results

### Consumption of *Blumeria graminis* Spores by Protists in Liquid Cultures

**Fig. 2:**
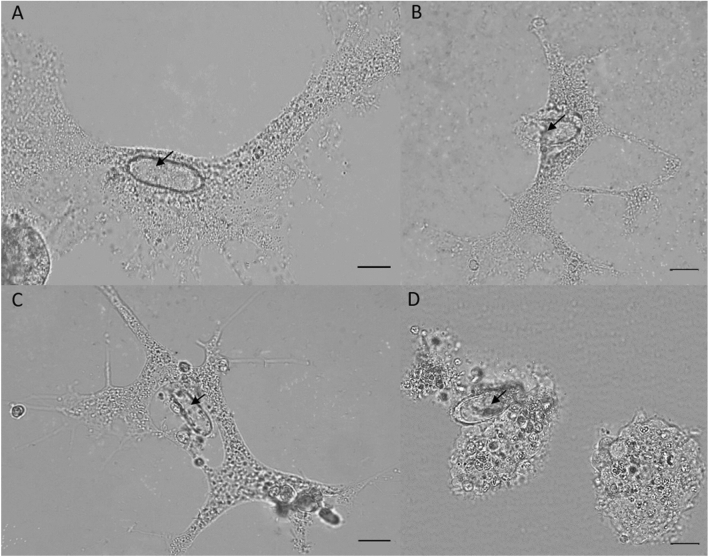
Interaction of the vampyrellid trophozoites with *Bgh*A6 spores in a liquid half-strength Waris-H culture. **a** Incorporation of one *Bgh*A6 spore (arrow) into the active trophozoite of the protist *L. vorax*. **b** Incorporation of one *Bgh*A6 spore (arrow) into the active trophozoite of *P. germanica*. **c** Incorporation of one *Bgh*A6 spore (arrow) into the active trophozoite of the protist *T. weberi* U11. **d** Docking of a *Bgh*A6 spore (arrow) to the contracted trophozoite of *T. weberi* G7.2. Scale bar = 10 µm

The five different cultures were first tested for their ability to incorporate the *Bgh*A6 spores. The *L. vorax*, *P. germanica*, *T. weberi* U11 and *T. weberi* G7.2 trophozoites (Fig. 2) were able to ingest the *Bgh*A6 spores and produced active trophozoites. At the beginning of the experiment, a high amount of *Bgh*A6 spores and a low amount of trophozoites and several cysts were seen. The first ingestion of some *Bgh*A6 spores into the trophozoites could be seen in the *L. vorax* culture. After seven days, the number of *Bgh*A6 spores was reduced strongly and the number of *L. vorax* trophozoites and digestive cysts increased. Without any refreshment, a culture with active trophozoites could be observed for a period of seven days. After the vast majority of the reserves have been used up, the number of trophozoites decreased while that of cysts increased. Inside the trophozoites and digestive cysts, the spores did not show any signs of growth, while the non-incorporated spores started a formation of spore chains. In a slightly staggered chronological order, the same effect was seen for the cultures *P. germanica*, *T. weberi* U11 and *T. weberi* G7.2, dependent on the natural reproduction rate. The *A. castellanii* culture did not interact with the *Bgh*A6 spores in the liquid culture. Instead, the trophozoites disappeared and the number of cysts increased strongly.

### Evaluation of the Powdery Mildew Colonies on the Golden Promise Leaves after Five Days

**Fig. 3:**
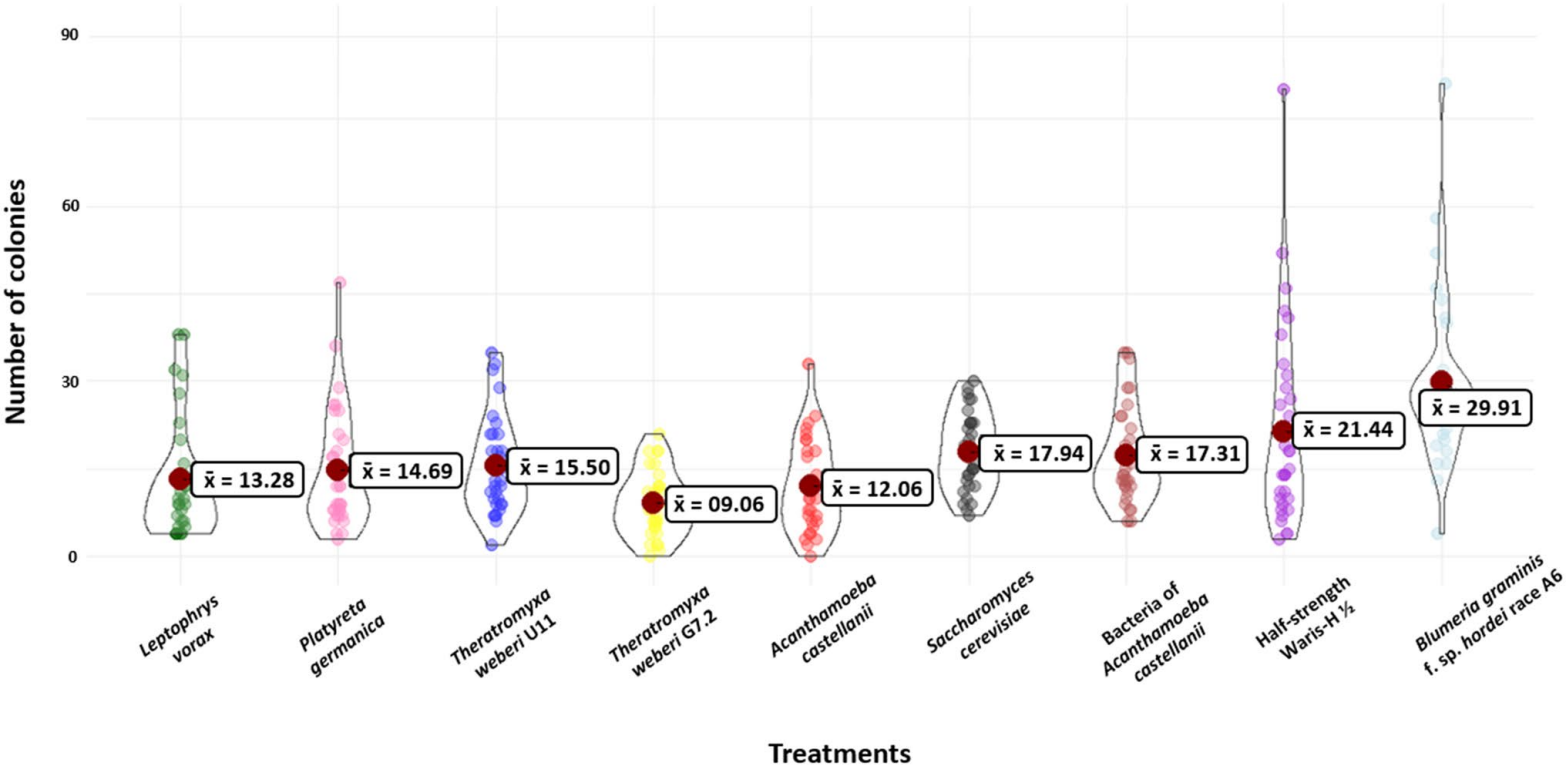
Violin plots of the total number of *Bgh*A6 colonies counted on the Golden Promise barley leaves of the different treatments. Each replicate of a single treatment is a point (32 replicates). Red points mark the mean value x̄

The number of powdery mildew colonies on the barley leaves showed a variable distribution of the 32 replicates of each treatment with several outliers (Fig. 3). All amoeboid treatments (1) *L. vorax*, (2) *P. germanica*, (3) *T. weberi* U11, (4) *T. weberi* G7.2 and (5) *A. castellanii* had smaller mean values compared to the four controls (6) *S. cerevisiae*, (7) Bacteria of *A. castellanii*, (8) half-strength Waris-H medium and (9) *Bgh*A6 only. The (4) *T. weberi* G7.2 treatment had the smallest median and mean value (x̄ = 9.1, X_Med_ = 8.5), while the (9) *Bgh*A6 treatment had the largest median and mean value (x̄ = 29.9, X_Med_ = 27.5).

Non-parametric Kruskal–Wallis statistical test showed significant differences between the treatments (*P* value = 9.3 × 10^–12^, *P* < 0.05). In addition, the post hoc pairwise Wilcoxon rank sum test also showed several significant differences between different treatments and their respective controls. Non suitable controls were not analysed (Table 2). *T. weberi* G7.2 and *A. castellanii* showed significant differences in the number of *Bgh*A6 colonies on the barley leaves compared to all of their respective controls, while the *L. vorax*, *P. germanica* and *T. weberi* U11 treatments did not. The *A. castellanii* treatment showed significantly fewer *Bgh*A6 colonies on the surface of the barley leaves compared to the control with the bacteria from the original culture, to the control with the media and to the control with the *Bgh*A6 spores only. The *T. weberi* G7.2 treatment also showed significantly fewer *Bgh*A6 colonies on the surface of the barley leaves compared to the *S. cerevisiae* control, to the media, and to the *Bgh*A6 spore control. *L. vorax* and *P. germanica* showed significantly fewer *Bgh*A6 colonies on the surface of the barley leaves compared to the *S. cerevisiae* and to the *BghA6* spore control. The treatments did not show significantly less *Bgh*A6 colonies on the surface of the barley leaves compared to the control with the media. *T. weberi* U11 showed significantly fewer *Bgh*A6 colonies on the surface of the barley leaves compared to the *Bgh*A6 spore control. Compared to the control with the *S. cerevisiae* and the control with the media, the treatment did not show significantly less *Bgh*A6 colonies on the surface of the barley leaves. Furthermore, the *T. weberi* G7.2 treatment showed significantly fewer *Bgh*A6 colonies on barley leaves compared to the treatments *P. germanica* and *T. weberi* U11 (Table 3 and Fig. 3), while the other comparisons showed no significant differences.

**Table 2:** Adjusted *P* values from the post hoc pairwise Wilcoxon rank sum test (*P* < 0.05) on the consumption activity of the *L. vorax*, *P. germanica*, *T. weberi* U11, *T. weberi* G7.2 and *A. castellanii* cultures on *Bgh*A6 spores on the surface of Golden Promise barley leaves

	(6) *S. cerevisiae*	(7) Bacteria of *A. castellanii*	(8) Half-strength Waris-H	(9) *Bgh*A6
(1)* L. vorax*	0.009**	–	0.052	2.1 × 10^–05^**
(2) *P. germanica*	0.043*	–	0.155	2.8 × 10^–05^**
(3) *T. weberi* U11	0.155	–	0.401	3.4 × 10^–05^**
(4) *T. weberi* G7.2	2.1 × 10^–05^**	–	0.002**	1.7 × 10^–08^**
(5) *A. castellanii*	–	0.030*	0.038*	8.5 × 10^–07^**

Pairwise comparison of the treatments with the different controls consisting of the food source *S. cerevisiae* or the bacteria of the *A. castellanii* culture, the medium half-strength Waris-H and the *Bgh*A6 spores only. *P < 0.05; **P < 0.01; – Treatment not performed

**Table 3:** Adjusted *P* values from the post hoc pairwise Wilcoxon rank sum test (*P* < 0.05) on the consumption activity of the cultures *L. vorax*, *P. germanica*, *T. weberi* U11, *T. weberi* G7.2 and *A. castellanii* on *Bgh*A6 spores on the surface of the barley leaves Golden Promise

	(1)* L. vorax*	(2) *P. germanica*	(3) *T. weberi* U11	(4) *T. weberi* G7.2
(1)* L. vorax*	–	–	–	–
(2) *P. germanica*	0.532	–	–	–
(3) *T. weberi* U11	0.152	0.400	–	–
(4) *T. weberi* G7.2	0.241	0.048*	0.003**	–
(5) *A. castellanii*	0.924	0.461	0.155	0.176

Pairwise comparison of the different treatments. *P < 0.05; **P < 0.01; – Treatment not performed

The datasets generated during and/or analysed during the current study are available from the corresponding author upon reasonable request.

### Viability Test of Inoculated Protists

**Fig. 4:**
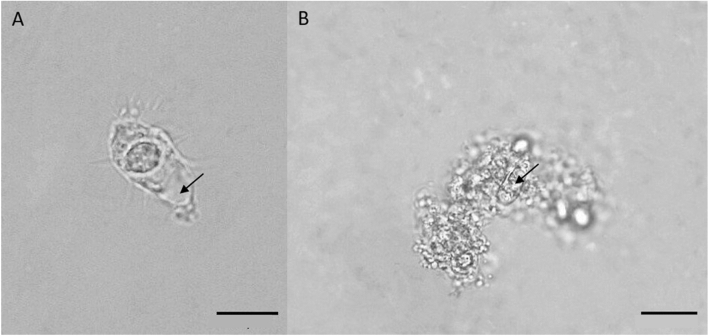
Liquid subcultures from the experiment in half-strength Waris-H. **a** Subculture of a piece of a leaf from the *A. castellanii* treatment, presumably after the consumption of a *Bgh*A6 spore (arrow). **b** Subculture of a piece of gel from the *T. weberi* G7.2 treatment detaining one *Bgh*A6 spore (arrow)

The subcultures were prepared to check protist survival over five days at experimental conditions. For this purpose, the leaves and pieces of agar were examined under the microscope 2 days after preparation. *L. vorax* showed several cysts in the liquid cultures, as did *P. germanica* and *T. weberi* U11. The treatments *A. castellanii* and *T. weberi* G7.2 showed some cysts as well as active cells in the cultures in contact with the *Bgh*A6 spores (Fig. 4). In particular, the *A. castellanii* culture from the leaf material showed larger (approx. 20 µm) and deformed trophozoites, caused by the possible consumption of a fungal spore (Fig. 4a).

### Spraying of Amoeboid Protists and Microscopy Evaluation

The culture, which was sprayed with a brown glass sprayer, was found to be partially lysed and had a battered appearance. The cysts of the *A. castellanii* culture appeared normal and unchanged, but the trophozoites were torn and damaged. The culture sprayed with the test tube sprayer had a better appearance. The cysts were unchanged and a few trophozoites could be detected, some were intact and active and some were ruptured.

## Discussion

The liquid culture experiment was designed to demonstrate the possibility of amoeboid protists feeding on the spores of the obligate biotrophic fungal pathogen and causative agent of powdery mildew disease of cereals, *Blumeria graminis*. Several studies showed that vampyrellids are able to engulf and consume fungal spores [13, 24–26], but this has not been shown with *Blumeria graminis* f. sp. *hordei (Bgh)* spores so far. The isolates *L. vorax*, *P. germanica*, *T. weberi* U11 and *T. weberi* G7.2 were able to incorporate the *Bgh*A*6* spores (Fig. 2). In contrast, the *A. castellanii* isolate did not clearly incorporate *Bgh*A*6* spores in the liquid culture. While the vampyrellid amoebae have mostly been studied for their pest control potential [22, 23, 25], *A. castellanii* is usually fed with a bacterial food source [39–42] and not used for fungal feeding experiments, because they capture their prey by phagocytosis and are therefore not expected to be able to handle larger prey. However, the incorporation and growth of *Acanthamoeba* fed on the yeasts *Saccharomyces cerevisiae* and *Cryptococcus laurentii* has already been demonstrated [29]. *A. castellanii* is also able to feed on the yeasts *Paracoccidioides* spp. and *Cryptococcus neoformans* [43, 44]. However, it is also known that *C. neoformans* has evolved adaptations to protect itself against its predator: when phagocytosed by *A. castellanii*, *C. neoformans* is able to replicate inside the predator’s cell, leading it to its death [44]. The same protective mechanism has been observed in the fungus *Aspergillus fumigatus* [45]. In contrast, the ingestion of the spores of the fungus *Fusarium culmorum* and subsequent protist growth was not possible [29]. Perhaps, as already observed for hyphae [29], the *Bgh*A6 spores were too large to be phagocytosed by *A. castellanii,* and there was no secondary consumption pattern as in vampyrellid amoebae [14, 15]. Despite these observations in the liquid culture experiment, all isolates were able to influence the *Bgh*A6 spores within five days and to survive on the barley’s leaf surface (Fig. 3). The protists *L. vorax*, *P. germanica* and *T. weberi* U11 showed cysts in the subcultures and the re-isolates *A. castellanii* and *T. weberi* G7.2 showed cysts and incorporated *Bgh*A6 spores after the experiment (Fig. 4). It is particularly interesting that *A. castellanii* was able to incorporate the *Bgh*A6 spores on the leaf surface: the trophozoites of the culture survived and were able to consume the spores (Fig. 4a), contrary to the results of the liquid culture experiment and previous knowledge. This finding requires further and more intensive research to understand the mechanisms behind the phagocytosis of the large spore, especially since a repetition of the experiment showed the same results (Table S3). The number of mildew colonies on the leaves confirmed the activity of the protist isolates, as all treatments with isolates showed significantly fewer mildew colonies compared to the control with *Bgh*A6 spores only. The isolate *T. weberi* U11 was significantly different from the control with *Bgh*A6 spores only, but not from the controls with the half-strength Waris-H medium and *S. cerevisiae*. However, it is not possible to determine exactly whether the observed effect is caused by the *T. weberi* U11, by the residual food source *S. cerevisiae* or the medium. On the one hand, the remaining food source *S. cerevisiae* could affect the *Bgh*A6 spores. It was shown that extracts from *S. cerevisiae* could control powdery mildew on barley by disrupting the fungal penetration [46]. Furthermore, it was suggested that *S. cerevisiae* is a potential alternative to chemical fungicides in the grape production for the prevention and control of powdery mildew [47]. There are also a number of products on the market that use *S. cerevisiae* to control powdery mildew (e.g. Romeo®, Intrachem Bio Deutschland GmbH & Co. KG, Germany). On the other hand, the liquid medium could interfere with fungal penetration by washing away the *Bgh*A6 spores. The *L. vorax* and *P. germanica* isolates showed significantly fewer powdery mildew colonies than the *Bgh*A6 spores-only control and the *S. cerevisiae* control. This means that the residual food source *S. cerevisiae* most likely did not cause the measured effect. In spite of this, there was no significant difference compared to the control with half-strength Waris-H. This means that the effect could be caused by the half-strength Waris-H medium by washing off the spores. The two treatments *A. castellanii* and *T. weberi* G7.2 showed significant differences in comparison to all of their controls with the food source *S. cerevisiae* or the bacteria of *A. castellanii*, with the half-strength Waris-H media and the *Bgh*A6 spores only. Therefore, the effect is most likely caused by the treatments and not by any other kind of interference. Combined with the results of the post-experiment subcultures, where active trophozoites were found in both cultures, it is most likely that the cells of these treatments were able to consume various attached microorganisms and many of the *Bgh*A6 spores on the barley leaves (Fig. 3). Again interestingly, *A. castellanii* was able to feed on the *Bgh*A6 spores from the leaves, something that had not been demonstrated in the liquid culture experiment or in the literature. Perhaps because there was no other viable food source in sufficient quantity, the pressure to survive led to the consumption of *Bgh*A6 spores. Furthermore, *T. weberi* G7.2 showed significantly fewer powdery mildew colonies on the barley leaves compared to the *P. germanica* and *T. weberi* U11 treatments. This suggests a possible use of the two fungus-consuming protists *A. castellanii* and *T. weberi* G7.2 as controlling agents of fungal infections by powdery mildew in the organic farming to optimise crop production, like it was already tried with the vampyrellid amoeba and the plant-damaging fungi *Gaeumannomyces graminis* [26]. However, others also tested *T. weberi* as a biological control agent and found that the trophic stages were not present fast enough due to the slow spread rate [21, 48]. Additional exclusion criteria such as drought susceptibility and non-specific feeding behaviour have to be considered before an application of *T. weberi* as a biological control agent, since the trophozoites had less drought stress in this experimental design, as they would have on normal leaves of a plant over a longer period of time. Nevertheless, in this experiment, the protists showed good potential for an environmentally friendly pest control product and the additional spraying test showed that further experiments would be useful. For this reason, *T. weberi* G7.2 and *A. castellanii* are optimal candidates for more intensive research into the control of powdery mildew on leaf surfaces, as they significantly reduced the number of mildew colonies and survived well on the barley leaf surface. This offers the prospect of reducing chemical fungicide use and possibly improve biological pathogen control for barley plants.

## Conclusion

All five protist isolates (*L. vorax*, *P. germanica*, *T. weberi* U11, *T. weberi* G7.2 and *A. castellanii*) were able to consume the *Bgh*A6 spores. The best results were obtained with the isolates *T. weberi* G7.2 and *A. castellanii*, which showed a significant reduction of powdery mildew colonies on the barley leaf surface compared to their respective controls. Based on several previous experiments and the results obtained in this study, further research is needed to confirm whether the two isolates *T. weberi* G7.2 and *A. castellanii* have potential for use as biological control agents against fungal infections with powdery mildew. Further studies could focus on the application procedure of protists on plants and their survival on the leaf surface and further experiments on plants in the greenhouse will give insights on spread rates of protists and susceptibility to drought [21].

### Supplementary Information

Below is the link to the electronic supplementary material.

Supplementary file1 (DOCX 29 KB)

## Data Availability

Data, material and codes can be provided upon request.

## Declarations

*Hordeum vulgare* cv. Golden Promise plants and the *Blumeria graminis* f. sp. *hordei* race A6 fungi were utilized.
