# Supplementary material for: Control of Blumeria graminis f. sp. hordei on Barley Leaves by Treatment with Fungi-Consuming Protist Isolates
Source: Curr Microbiol. 2023 Oct 23;80(12):384. doi: 10.1007/s00284-023-03497-5 (PMC10593611; doi:10.1007/s00284-023-03497-5)
Supplement: Supplementary file 1 — Supplementary file1 (DOCX 29 KB) [file 284_2023_3497_MOESM1_ESM.docx]

**Supplementary data**

**Control of *Blumeria graminis* f.sp. *hordei* on barley leaves by treatment with fungi-consuming protist isolates**

Julia Sacharow ^a^, Elnaz Salehi-Mobarakeh ^a^, Stefan Ratering ^a^, Jafargholi Imani ^b^, Alessandra Österreicher Cunha-Dupont ^a^ and Sylvia Schnell ^a^

^a^ Institute of Applied Microbiology, IFZ, Justus-Liebig-University Giessen, Germany

^b^ Institute of Phytopathology, IFZ, Justus-Liebig-University Giessen, Germany

**Tab. S1:** Pre-experiment with different inoculation procedures and different concentrations of *Blumeria graminis* spores (10^2^, 10^4^ and 10^5^) on barley leaves. Results of eight replicates.

| Procedure | Description | Results |
| --- | --- | --- |
| Suspension inoculation | Four leaves were placed in a reaction tube with 40 ml *Blumeria graminis* spore solution for 30 min at 125 RPM and 25 °C. | On one leave two colonies were counted (concentration 10^5^). |
| Pipette inoculation | 100 µl *Blumeria graminis* spore solution were dripped on one leaf and distributed with a Drigalski spatula. | No colonies were counted. |
| Spraying inoculation | On one leaf 1 ml of *Blumeria graminis* spore solution was sprayed. | On one leave two colonies were counted (concentration 10^5^). |

**Tab. S2:** Pre-experiment with half-strength Waris-H application on the barley leaves before and after the *Bgh*A6 spore inoculation. Each treatment has eight replicates (total number). Results are separated in small, big and total *Bgh*A6 colony numbers.

|  | Total number of small colonies | Total number of big colonies | Total number of colonies |
| --- | --- | --- | --- |
| *Bgh*A6 only | 47 | 38 | 85 |
| Half-strength Waris-H (before *Bgh*A6) | 27 | 31 | 58 |
| Half-strength Waris-H (after *Bgh*A6) | 25 | 42 | 67 |

**Tab. S3:** Repetition of the experiment with the *A. castellanii* inoculation, 60 min after the inoculation with the *B. graminis* spores. Each treatment has eight replicates (total number).

|  | Total number of colonies |
| --- | --- |
| *Bgh*A6 only | 90 |
| Half-strength Waris-H | 67 |
| *A. castellanii* | 1 |
